# The Over-Expression of *E2F3* Might Serve as Prognostic Marker for Neuroblastoma Patients with Stage 4S Disease

**DOI:** 10.3390/diagnostics10050315

**Published:** 2020-05-16

**Authors:** Stefano Parodi, Marzia Ognibene, Riccardo Haupt, Annalisa Pezzolo

**Affiliations:** 1 U.O. Epidemiologia e Biostatistica, IRCCS Istituto Giannina Gaslini, 16147 Genova, Italy; riccardohaupt@gaslini.org; 2 U.O.C. Laboratorio Cellule Staminali Post Natali e Terapie Cellulari, IRCCS Istituto Giannina Gaslini, 16147 Genova, Italy; marziaognibene@gaslini.org

**Keywords:** neuroblastoma, stage 4S disease, E2F3 protein, prognostic marker

## Abstract

Stage 4S neuroblastoma is a childhood cancer occurring in infants (<12 months at diagnosis) with metastases limited to liver, skin, and bone marrow (<10%). It is associated with an excellent outcome, due to its notable ability to undergo spontaneous regression without any therapeutic intervention. However, a subgroup of patients is doomed to relapse and eventually to die in spite of aggressive therapies. Stage 4S neuroblastoma shows characteristic hypermethylation of genes involved in the telomere maintenance, indicating that the dysregulation of these genes might serve as prognostic marker. The retinoblastoma tumor suppressor protein (RB)-E2F transcription factors pathway is one of the critical tumor-suppressor/oncogene pathways involved in regulating telomerase expression. We have interrogated in silicopublic neuroblastoma databases for regulators involved in the RB-E2F pathway especially for E2F factors themselves, and we identified the E2F transcription factor 3 (E2F3) expression as a potential prognostic marker in stage 4S neuroblastoma. In order to confirm this finding, we screened 38 paraffin-embedded tissue samples stage 4S neuroblastoma for E2F3 protein expression using immunofluorescence, and we observed that augmented expression was strongly associated with impaired event-free survival. These results indicate that E2F3 expression might serve as prognostic marker in patients with stage 4S disease.

## 1. Introduction

Neuroblastoma (NB) is the most common extra-cranial solid tumor of childhood, and it is the main cause of death in children between 1 and 4 years [1,2]. NB is considered a developmental disorder resulting from the interruption of normal sympathetic neuronal progenitor maturation [3]. NB is a heterogeneous disease with prognosis ranging from long-term survival to fatal outcome [4]. The clinical and biological parameters are used for therapeutic stratification of NB patients [5,6]. Stage 4S NB is a special type of NB established in infants with metastases limited to the liver, skin, and bone marrow (<10%) at diagnosis and is associated with an excellent outcome due to its notable ability to undergo spontaneous regression without any therapeutic intervention [7]. Unfortunately, a subgroup of patients with stage 4S disease has worse outcomes. Prognostic factors at diagnosis include *MYCN* amplification, recurrent segmental chromosomal aberrations (losses of chromosome arms 1p, 3p, 4p, 6q, 11q and gains of chromosome arms 1q, 2p, 17q), diploid DNA index, age younger than 4 weeks, and life-threatening symptoms [8,9,10,11,12]. Thus, it is imperative to identify new therapeutic targets and to establish differentiation inducing protocols. The exact mechanisms responsible for spontaneous regression or differentiation into a benign ganglioneuroma without treatment are unknown. Several possible mechanisms have been proposed to explain spontaneous regression: neurotrophin deprivation, loss of telomerase activity, cellular or humoral immunity, and alterations in epigenetic regulation [13,14,15]. It has been demonstrated that the DNA methylation pattern of stage 4S NB is characterized by differential methylation of target genes of transcription factors involved in neural crest development and neuronal differentiation [13,14]. The DNA methylation portrait is anew mechanism that may contribute to the stage 4S tumor progression or spontaneous regression. The *telomerase reverse transcriptase* (*TERT*) gene encoding the catalytic subunit of telomerase involved in telomere length regulation [15] and its promoter [16] are hypermethylated in NB samples from patients with stage 4S disease [13]. Interestingly, stage 4S tumors showed characteristic hypermethylation of genes involving in an important pathway the retinoblastoma tumor suppressor protein (RB)-E2F oncogene transcription factors [17]. The RB-E2F pathway is one of the critical tumor-suppressor/oncogene pathways involved in regulating *TERT* gene expression [17]. It has been demonstrated that *TERT* is expressed at lower levels in stage 4S compared to stage 4 NB and that low TERT activity or short telomeres might be associated with spontaneous regression of this special type of NB [14,18]. The *retinoblastoma tumor suppressorRB1* gene encodes for a protein pRB that acts as a tumor suppressor regulating cell growth and keeps cells from dividing too fast or without control [19]. Inactivation of RB1 expression in tumor cells leads to the deregulation of activity of transcription factors E2F1, E2F2, and E2F3, which control the expression of genes involved in differentiation, development, proliferation, and apoptosis [20,21,22,23,24]. To notice, the transcription factors E2F1, E2F2, and E2F3 bind to the proximal *MYCN* promoter, specifically in NB cell lines expressing *MYCN* [21]. Several transcription factors that are clue in normal neuronal development, as well as the cell cycle regulator E2F3, were found to be up-regulated in a murine model of human MYCN-driven NB [22]. E2F3 is part of the E2F family of transcription factors that includes eight members (E2F1-8) [20]. It has been suggested that miR-34a could have a role as tumor suppressor in NB tumorigenesis by directly binding to *E2F3* mRNA and significantly reducingthe level of E2F3 protein [25]. However, no studies have examined the role of RB-E2F pathway in stage 4S NB. The expression of *RB1* gene may be responsible for the block of cell cycle progression and decreased TERT activity in stage 4S undergoing spontaneous regression. A crucial goal should be to determine whether the over-expression of one or more of the genes involved in RB-E2F pathway and of *TERT* gene might serve as prognostic markers for patients with stage 4S with worse outcomes. Here, we have examined in silicothree public NB databases from R2 platform for *RB1*, *E2F1*, *E2F2*, *E2F3*, and *TERT* gene expressions.

## 2. Results

### 2.1. Association of RB1, TERT, E2F1, E2F2, and E2F3 Gene Expressionswith Clinical Outcome in Stage 4S Neuroblastoma Patients

**Figure 1:**
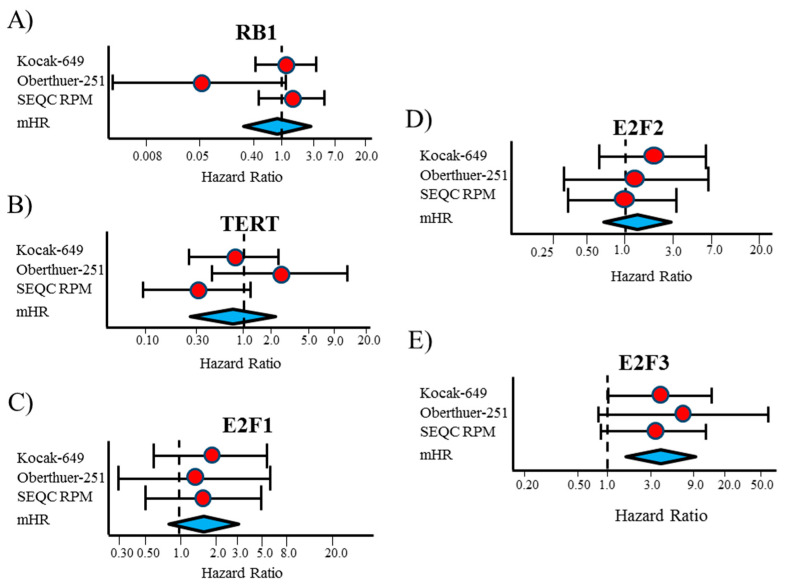
Forest plot of hazard ratios for the association between Event Free Survival of patients with Stage 4S neuroblastoma and the considered gene expressions, categorized on the basis of the median expression value. (**A**) *RB1*; (**B**) *TERT*; (**C**) *E2F1*; (**D**) *E2F2*; (**E**) *E2F3*. mHR = meta-analytic estimate of HR.

We examined how *RB1*, *TERT*, *E2F1*, *E2F2***,** and *E2F3* gene expressions linked to event-free survival (EFS) in stage 4S NB patients using gene expressions in the publicly available datasets consisting of primary tumor samples from three independent NB patient cohorts (Kocak-649 [26], Oberthuer-251 [27], and SEQC-RPM [28] datasets), downloaded from the R2: Genomic Analysis and Visualization Platform (available online: http://r2.amc.nl). The three data sets included microarray expression profiles of 134 stage 4S NB with *MYCN* normal status and 32 events, defined as disease relapse, disease progression, or death for any cause. In more details, Kocak-649 included 78 stage 4S NB patients, of which 17 were excluded for lack of information on patients’ survival, 4 for *MYCN* amplification, and 1 for missing data about *MYCN* status, thus leaving 56 patients (13 events) available for the analyses. Oberthuer-251 included 31 patients and among these one with *MYCN* amplification; then, the analyses were carried out on 30 patients (7 events). Finally, SEQC-RPM, included 53 patients, of which 5 were excluded for *MYCN* amplification (*n* = 4) or missing data for *MYCN* status (*n*=1), leaving 48 patients available for the analyses (12 events). The SEQC-custom data set, which contains information from the same samples of SEQC-RPM, profiled by a different microarray platform, was analyzed separately. For each gene, patients were split into two groups of the same size, using the median value of gene expression. Sensitivity analysis was carried out by splitting the patients on the basis of two different cut-offs (the first and the last tertile of the expression values distribution). Figure 1A–E shows the results of EFS analysis as a forest plot, obtained displaying the hazard ratio (HR) estimates for each gene, the corresponding meta-analytic HR estimate (mHR), and their related 95% Confidence Intervals (95% CI). The corresponding values are reported in Table 1.

For *RB1* gene expression, a large heterogeneity was observed between the three considered data sets (Figure 1A). The corresponding mHR was close to one (0.87, 95% CI: 0.27–2.8), the expected value under the null hypothesis of no association between gene expression and patient’s survival (Table 1). The corresponding Kaplan–Meier curves revealed a noticeable heterogeneity, with a poor survival rate in patients with low *RB1* expression in the Oberthuer data set, but not in the other databases (Figure S1). A similar pattern also emerged in the sensitivity analysis at two different cut-offs (Figures S2–S4).

No association was observed between *TERT* gene expression and EFS (mHR = 0.80, 95% CI: 0.29–2.2, Figure 1B and Table 1). Analysis at each selected cut-off did not point out any prognostic role of *TERT* expression (Figures S5–S8), even if in the SEQC-RPM data set a poorer survival was observed for patients with expression values lower than the median (Figure S5C) and the first tertile (Figure S6C), but without achieving statistical significance.

A low *E2F1* expression was slightly associated with a better EFS in the three considered data sets and, accordingly, in the meta-analytic analysis, even if statistical significance was not reached (mHR = 1.6, 95% CI: 0.80–3.3, Figure 1C and Table 1). A higher EFS was consistently observed for patients with low *E2F1* gene expression in each considered data set and for each selected cut-off (Figures S9–S11), which was statistically significant when patients were categorized on the basis of the first tertile (mHR = 3.0, 95% CI: 1.2–7.8, Figure S12A).

High *E2F2* expression was slightly associated with a poor EFS in all considered data sets, except SEQC-RPM (Figure 1D and Figure S13), but statistical significance was not reached (mHR = 1.4, 95% CI: 0.70–2.9, Table 1). In the sensitivity analysis (Figures S14–S16), this association seemed to be slightly stronger when the first tertile of the gene distribution was selected as cut-off.

**Figure 2:**
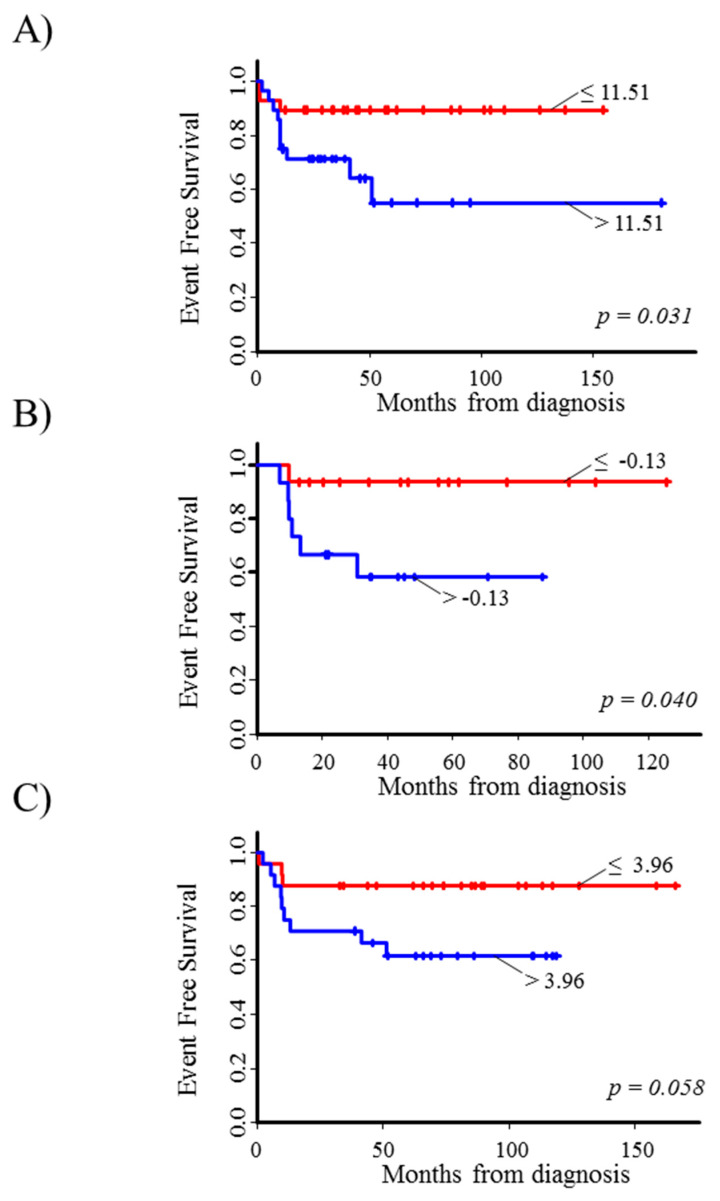
Event Free Survival curves of stage 4S neuroblastoma patients in relation to *E2F3* gene expression levels in the three considered microarray datasets. (**A**) Kocak-649; (**B**) Oberthuer-251; (**C**) SEQC-RPM. Patients were split into two groups (red line: lower values; blue line: higher values) on the basis of the median value of gene expression (reported near the corresponding curve).

Finally, high levels of *E2F3* gene expression were associated to a poor survival in each considered data set (Figure 1E, and Figure 2A–C). The corresponding mHR was 3.9 (95% CI: 1.7–9.1, *p* = 0.002, Table 1). Results from sensitivity analysis consistently confirmed the association observed at each selected cut-off (Figures S17–S20).

The role of E2F3 in the prognosis of stage 4S NB patients with *MYCN* amplification is worthy of further investigations. Unfortunately, in the data sets analyzed stage 4S NB patients with tumor *MYCN* amplified are only 9 (4 in Kocak-649, 1 in Oberthuer-251, and 4 in SEQC-RPM database respectively), thus preventing survival analysis from being performed.

### 2.2. E2F3 Protein Expression in Primary Stage 4S Neuroblastoma Tissue Sections

NB paraffin-embedded tissue sections of NB tumors from 38 patients with stage 4S disease were tested for E2F3 protein expression by immunofluorescence. Twenty-four patients did not have relapse or tumor progression after a median follow up time of 2.9 months (range: 9 days–18.3 months). One patient relapsed at a local site, and 13 experienced distant metastases. These included 4 stage 4 sites (namely: 1 lung, 1 central nervous system, and 2 bones, who also included bone marrow in both and liver in one), 3 distant and local sites combined, and 6 metastatic sites only. Median follow up time among non-relapsed patients was 109 months (range: 43.3–187.1 months). All patients, but 5 (3 non relapsed and 2 relapsed), had a normal *MYCN* status.

**Figure 3:**
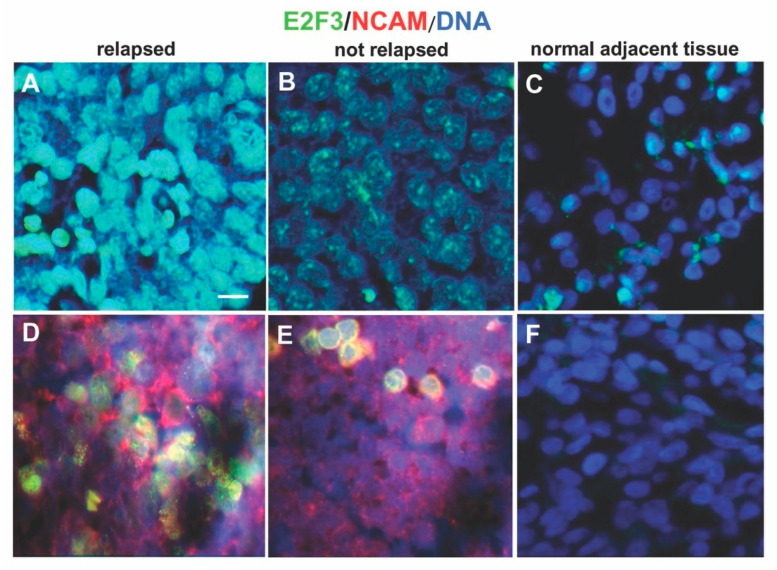
Expression of E2F3 protein. Immunofluorescence assay was performed to test the expression of E2F3 protein in neuroblastoma 4S tissues using the anti-E2F3 antibody. (**A**) Representative image showing brilliant green nuclear staining for E2F3 present in one specimen out of the 14 relapsed patients analyzed. (**B**) Representative image presenting weak green nuclear staining of E2F3 detected in one sample from not relapsed patients. (**C**) E2F3 protein expression in normal adjacent tissues. (**D**,**E**) Representative images of tumor cells (red) identified using the NB specific marker NCAM (CD56). (**F**) Normal adjacent tissues negative for NCAM. DAPI was used for DNA staining and is shown as blue. Three independent experiments were performed. Scale bars: 25 µm.

Brilliant and intense green nuclear staining for E2F3 was present in the specimens from relapsed patients indicative of strong expression. Weak green nuclear staining was detected in the samples from not relapsed patients indicative of low expression of E2F3 protein.The number of cells with intense nuclear staining for E2F3 protein was higher in tissues from primary stage 4S tumors that relapsed or progressed compared to not relapsed tumors showing weak intensity of E2F3 nuclear staining (*p* < 0.001, chi squared test) (Figure 3A,B and Table S1). For control, the proportion of cells with nuclear staining for E2F3 protein was higher in tissues from primary stage 4S NB than in normal adjacent tissues (E2F3^+^ nuclei 90 ± 3% vs. 10 ± 1%, *p* = 0.002) (Figure 3C).

## 3. Discussion

Patients with stage 4S disease (7%–10% of NB) generally have favorable outcomes, often exhibiting spontaneous maturation and regression, or needing moderate intensity chemotherapy, or low dose of radiotherapy to the liver [1,2,3,4,5,6]. Unfortunately, 10%–25% of patients with stage 4S NB do not survive, mostly due to either rapid progression of liver metastases, or development of hepatomegaly related to disease-causing compression of normal liver tissue, lungs, kidneys, inferior vena cava, and intestines [7,8,9,10]. Tumor progression to high risk stage 4 is observed occasionally [8]. Disease progression of stage 4S is strongly related to the presence of unfavorable prognostic biologic markers in the tumor and age younger than 4 weeks at diagnosis [8,9,10,11,12]. Stage 4S NB represents two biologically different sub-groups, one of which shows whole-chromosome numerical abnormalities typical of localized tumors (stages 1, 2, 3), whereas the other carries recurrent imbalance chromosomal structural rearrangements, as well as TERT activity, representative of clinically advanced tumors (stage 4) [29]. Notably, it has been suggested that stage 4S spontaneous regression is related to the loss of TERT activity and telomere shortening [14,18]. Our group examined the telomere length, TERT expression, and the presence of alternative lengthening of telomeres (ALT) mechanism in 100 NB samples; most of the 4S specimens had low TERT expression or short telomeres associated with a good prognosis [18]. A recent study found that NB patients whose tumors lacked telomere maintenance had an excellent prognosis, whereas patients whose tumors harbored TERT gene over-expression had worse outcome [30]. The aggressive stage 4 NB expressed high levels of TERT, whereas tumors with favorable outcome had no or little expression [30]. Spontaneous tumor regression without treatment occurred in 4S NB patients with no telomere maintenance [30]. It is likely that a better understanding of the mechanism(s) of tumor progression or spontaneous regression/differentiation will support the identification of targeted therapeutic approaches for stage 4S NB patients.

The RB-E2F axis is one of the critical tumor-suppressor/oncogene pathways involved in regulating *TERT* expression. Inactivation of *RB1* expression in cancer leads to the deregulated activity of the transcription of *E2F1*, *E2F2*, and *E2F3* [24].

Here we examined how *RB1*, *E2F1*, *E2F2*, *E2F3*, and *TERT* gene expressions linked to survival in stage 4S NB patients using microarray mRNA expression data from three independent public NB patient cohorts. We have identified that *RB1* and *TERT* gene expressions had no association with EFS; low *E2F1*, *E2F2* expression was slightly associated with a good EFS in all considered data sets, whereas high levels of *E2F3* gene expression were associated to a poor survival in each considered data set. E2F3 could be a new potential prognostic marker of stage 4S NB without *MYCN* amplification. In the data sets analyzed in this work, only 9 stage 4S NB patients presented *MYCN* amplified tumor; however, we could not analyze either the E2F3 expression or its relation with survival of this group of patients.

E2F3 is the major member of the E2F family protein and has an important role in regulating gene transcription, cell cycle, proliferation, and apoptosis [31,32]. It has been described that E2F3 protein regulated *MYCN* transcription and is required for full activity of the *MYCN* promoter in NB [21]. The up-regulation of the transcription factor encoded by the *MYCN* gene is essential for cell-cycle control, growth-factor dependence, apoptosis, and block of differentiation in primary neural crest cells [22]. Over-expression of E2F3 protein that accompanies activation of the *MYCN* gene might represent the driving factor of stage 4S NB progression.

It has been shown that elevated level of E2F3 protein drives ectopic proliferation in multiple tissues [33]. Interestingly, *E2F3* is an oncogene with strong proliferative potential, and it is over-expressed in multiple cancers [34,35,36,37]. Amplification or elevated expression of the *E2F3* locus at 6p22.3 has been identified in breast [34], prostate [35], bladder [36], and lung cancers [37]. Gain or amplification of chromosome 6p22.3 is not recurrent in NB [2,4,38], and *E2F3* gene expression is not activated bona fide through chromosomal abnormalities, therefore an oncogenic mechanism probably exists that operates independently of gene amplification. We postulate that still-unknown factors affect the level and timing of oncogene over-expression and determine the specific mechanism used by neuroblast to aberrantly express each oncogene. Moreover, cancer genome-wide association studies (GWAS) showed that the malignant transformation of developing neuroblast is influenced by common variations (polymorphisms) in the genome at 6p22.3 and that the NB phenotype is in part determined by germline variations at this locus [39,40]. These data suggest that the chromosome 6p22.3 risk alleles not only influence the developing NB but also the likelihood ofdeveloping a more malignant phenotype [39,40]. It is not known how the presence of common DNA variants at 6p22.3 locus contributes to the risk of neuroblastic malignant transformation, presumably through altered expression and/or alternative splicing of regional candidate genes. Here, we reported that the *E2F3* gene located at 6p22.3 appeared to play an oncogenic role in NB 4S stage. Using publicly available mRNA expression data, we have demonstrated that *E2F3* high expression is associated with reduced survival of NB patients with stage 4S disease. For further confirmation, we performed a retrospective investigation detecting a high level of nuclear staining for E2F3 protein in 4S tumors with the occurrence of relapse or progression, whereas the non-progressing tumors had low level of this protein, suggesting that E2F3 over-expression may represent an earlier event in NB progression. Genomic studies have demonstrated that tumors that amplify *E2F3* often also acquire mutations in RB1 with consequent inactivation of the RB pathway [41], but we did not find any association between *RB1* gene expression in stage 4S NB patients and clinical outcome. Our observation that nuclear E2F3 protein levels were elevated in primary tissues from stage 4S NB relapsed or progressed compared with tumors from stage 4S patients without any events, suggests that there may be continuous selection in the former tumors for unknown mechanisms that promote E2F3 activity. E2F3 may be subjected to further mechanisms of post-translation deregulation that are altered by oncogenesis. DNA methylation serves as a regulatory mechanism for transcription factor activity; therefore, we suggest that hypomethylation or common polymorphisms with enhancer activity influencing expression of protein coding gene at 6p22.3 might be the causes for the high expression of *E2F3,* but more studies need to be done. We postulate that 6p22.3 region becomes hypomethylated or that *E2F3* can be a susceptibility gene influenced by polymorphisms, lying within putative regulatory region, resulting in augmented *E2F3* expression associated with disease phenotype and NB 4S patient’s outcome. Therefore, augmented *E2F3* expression coming from an inherited polymorphism would impact neural crest cellular lineage commitment and predispose these cells to undergo malignant transformation. Additional studies are needed to develop more specific information about DNA methylation, histone modification, chromatin remodeling, or functional polymorphisms affecting gene expression during regression and differentiation of NB 4S tumors.

## 4. Materials and Methods

### 4.1. R2 Genomic Analysis and Visualization Platform

We analyzed the data of RNA microarray experiments from three independent datasets consisting of 134 NB 4S samples for *RB1*, *TERT*, *E2F1*, *E2F2*, *E2F3* gene expressions extracted from the R2 Genomics Analysis and Visualization Platform (http://r2.amc.nl, last downloading: December 20, 2019). Selection criteria included at least 10 tumour samples of Stage 4S Neuroblastoma, no *MYCN* amplification, availability of time-to-event information for survival analysis. Three R2 databases fulfilling the inclusion criteria were identified, namely, Kocak-649 [26], Oberthuer-251 [27], and SEQC-RPM [28]. Furthermore, the data set SEQC-custom, which included the same samples of SEQC-RPM profiled by a different platform was also considered and analyzed separately.

### 4.2. Immunofluorescence Analysis

Immunofluorescence analysis was performed on formalin-fixed, paraffin-embedded NB specimens (4 μm-thick) as previously described [42]. We used the mouse monoclonal antibody E2F-3 (D-2) (Santa Cruz Biotechnology, Dallas, TX, USA) and the CD56 (NCAM) polyclonal antibody (ThermoFisher Scientific, Waltham, MA, USA). We used isotype matched non-binding mAbs in all antibody staining experiments to avoid nonspecific reactivity. Results were photographically documented using fluorescence microscope Axio Imager M2 equipped with ApoTome System (Carl Zeiss, Oberkochen, Germany). For NB specimens, each tumor area tested contained malignant cells as assessed by histologic examination. Tumor cells were identified in each sample using the NB specific marker NCAM (CD56) [43] (Figure 3D,E). Quantification of immunofluorescence positive tumor cells was performed on serial tissue sections, thus allowing quantification in tumor areas selected by the pathologist. The proportion of immunofluorescence positive cells counted was at least 100 to 1000 cells and reported as percentage for the subsequent statistical analysis.

### 4.3. Patients and Tumor Samples

We recovered a multi-institution retrospective series of primary NB tissue sections from 38 patients with stage 4S disease, diagnosed in the period from December 2000 to October 2011 in 27 centers of the Italian Association of Pediatric Hematology and Oncology (AIEOP) [44]. The tumors tissue specimens are stored in the BIT-NB Biobank of IstitutoGianninaGaslini, Genova, Italy. The patient data derived from Italian Neuroblastoma Registry (INBR) of AIEOP. The clinical characteristics of NB patients are collected in pseudo-anonymized manner and stored in a secure system at the Epidemiology and Biostatistics Unit of the IstitutoGianninaGaslini. This prospective Study Registry is conducted according to the principles of the Declaration of Helsinki (59th WMA General Assembly, Seoul, October 2008) and all its revisions, pertinent national laws and regulations, as well as the International Conference on Harmonization’s Good Clinical Practice, taking into account the Directive 2001/20/EC of the European Parliament and of the Council April 2001. Medical records were abstracted at each institution, and clinical data including age at diagnosis, sex, stage, *MYCN* status, DNA index, histology, and outcome were collected. The patients were staged according to the International Neuroblastoma Staging System [6]. This study was conducted in accordance with the Declaration of Helsinki and approved by the Italian Institutional Ethics Committee (Measure n° 270/17 related to the clinical study protocol IGG-NCA-AP-2016). Written informed consent was obtained from all patients or their legal guardians.

### 4.4. Statistical Methods

Event Free survival (EFS) was evaluated by the Kaplan–Meier method, splitting the NB patients into two groups on the basis of the median value of each gene expression profile. A sensitivity analysis was also carried out using two different cut-offs (the first and the last tertile of the gene expression distribution). The corresponding Hazard Ratios (HRs) were estimated by Cox regression model, applying the Firth’s correction, based on a penalized likelihood function, in the presence of zero observed events in either one group [45]. For each considered gene, a meta-analytic common estimate of HR (mHR) and its related 95% Confidence Intervals (95% CI) were obtained using the random effect model by DerSimmonianandLeird (1986) [46], under the assumption of a log-normal distribution for both HR and mHR [47]. Survival analyses were also repeated by replacing the SEQC-RPM database with its homologous SEQC-custom, in order to check for the consistency of mHR estimates.

All analyses were performed by R statistical software, release 3.3.3 [48]. In detail, the R library *survival* was used to fit Cox regression models and to draw the corresponding Kaplan–Meier curves. The *coxphf* R library was employed to apply the Firth correction. Finally, an ad hoc *routine* in R language was implemented to estimate mHR and its related 95% CI.

## 5. Conclusions

In summary, interrogating in silicopublic NB databases, we found that high *E2F3* expression level strongly correlates with worse outcome of NB patients with stage 4S disease. Moreover, we screened primary tumor tissue from stage 4S NB for E2F3 protein expression using immunofluorescence, and we found that high level of nuclear E2F3 expression was strongly associated with disease relapse or progression.

Our results indicate that high expression of E2F3 can promote NB progression. The potential prognostic role of E2F3 in Stage 4S NB progression should be elucidated by extensive research.

## Figures and Tables

**Table 1:** Hazard ratios for the association between event free survival of patients with Stage 4S neuroblastoma and the considered gene expressions, categorized on the basis of the median expression value.

Data Set	N/E	HR	95% CI	*p*
*RB1*				
Kocak-649	56/13	1.2	0.39–3.4	*0.795*
Oberthuer-251	30/7	0.06	0.00–0.47	*0.003*
SEQC-RPM	48/12	1.4	0.45–4.5	*0.546*
mHR	134/32	0.87	0.27–2.8	*0.821*
*TERT*				
Kocak-649	56/13	0.82	0.27–2.4	*0.719*
Oberthuer-251	30/7	2.5	0.48–12.7	*0.266*
SEQC-RPM	48/12	0.33	0.09–1.2	*0.082*
mHR	134/32	0.80	0.29–2.2	*0.666*
*E2F1*				
Kocak-649	56/13	1.9	0.61–5.7	*0.268*
Oberthuer-251	30/7	1.3	0.30–6.0	*0.705*
SEQC-RPM	48/12	1.6	0.50–5.0	*0.429*
mHR	134/32	1.6	0.80–3.3	*0.177*
*E2F2*				
Kocak-649	56/13	2.0	0.64–6.0	*0.233*
Oberthuer-251	30/7	1.4	0.31–6.1	*0.675*
SEQC-RPM	48/12	1.0	0.34–3.2	*0.942*
mHR	134/32	1.4	0.70–2.9	*0.330*
*E2F3*				
Kocak-649	56/13	3.8	1.0–13.7	*0.031*
Oberthuer-251	30/7	6.8	0.81–56.2	*0.040*
SEQC-RPM	48/12	3.3	0.89–12.2	*0.058*
mHR	134/32	3.9	1.7–9.1	*0.002*

N/E = number of samples/number of events; HR = Hazard Ratio; 95% CI = 95% Confidence Intervals of HR; mHR = meta-analytic estimate of HR.

## Supplementary Materials

The following are available online at https://www.mdpi.com/2075-4418/10/5/315/s1.

## Abbreviations

NB	Neuroblastoma
TERT	telomerase reverse transcriptase
RB1	retinoblastoma tumor suppressor
EFS	event-free survival
HR	hazard ratio
mHR	meta-analytic HR
ALT	alternative lengthening of telomeres
*MYCN*	v-mycmyelocytomatosis viral related oncogene neuroblastoma derived
GWAS	genome-wide association studies
INSS	International Neuroblastoma Staging System
INBR	Italian Neuroblastoma Registry
SIOPEN	International Society of Paediatric Oncology European Neuroblastoma
AIEOP	Italian Association of Pediatric Hematology and Oncology
