# Supplementary material for: The Over-Expression of E2F3 Might Serve as Prognostic Marker for Neuroblastoma Patients with Stage 4S Disease
Source: Diagnostics (Basel). 2020 May 16;10(5):315. doi: 10.3390/diagnostics10050315 (PMC7277942; doi:10.3390/diagnostics10050315)
Supplement: Supplementary file 1 [file diagnostics-10-00315-s001.pdf]

The over-expression of *E2F3* might serve as prognostic marker for neuroblastoma patients with stage 4S disease

Supplemental Material

Figure S1. Event Free Survival of Stage 4S Neuroblastoma patients in relation to the expression of *RB1* gene in four microarray databases; cut-off value based on the median of gene expression (red line: lower values; blue line: higher values).
Panel A: Kocak-649 data set; panel B: Oberthuer-251 data set; panel C: SEQC dataset, RPM platform; Panel D: SEQC data set, custom platform.

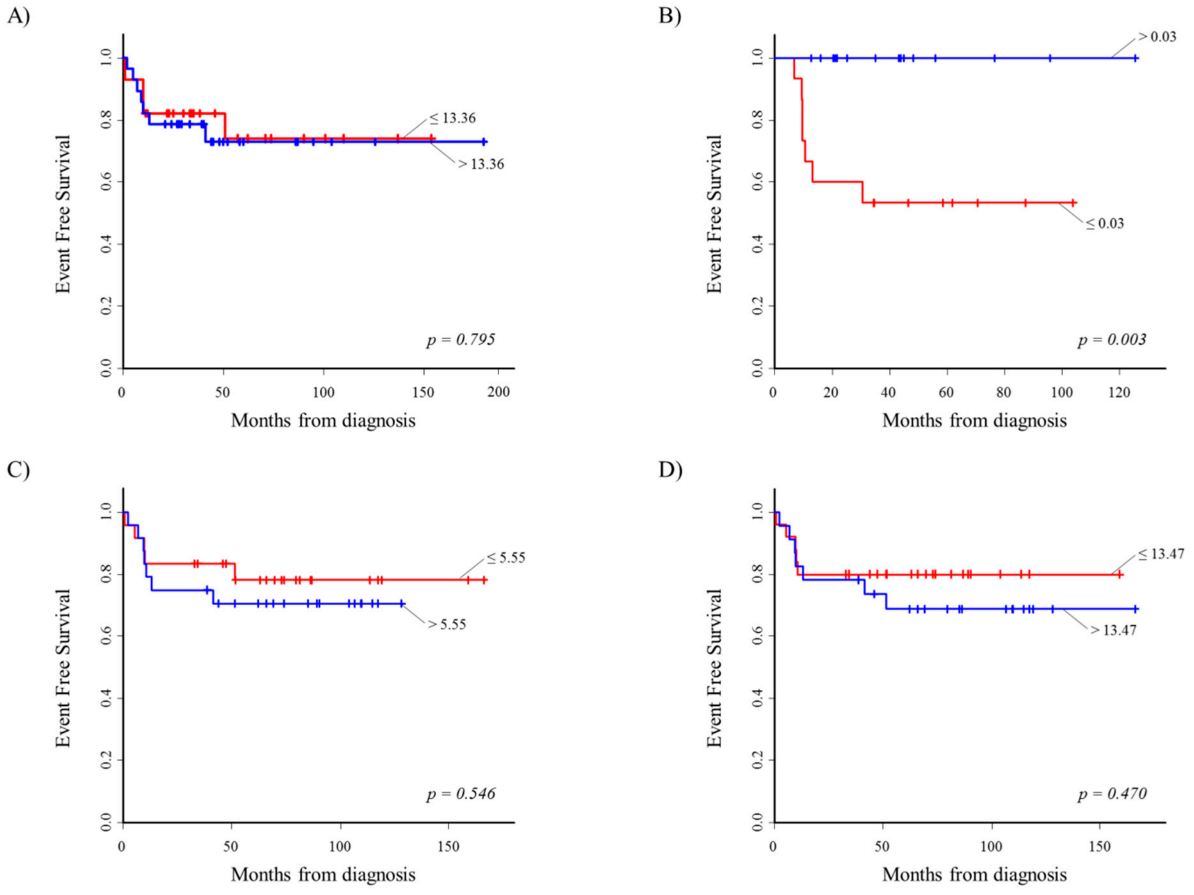

Figure S2. Event Free Survival of Stage 4S Neuroblastoma patients in relation to the expression of *RB1* gene in four microarray databases; cut-off value based on the first tertile of gene expression (red line: lower values; blue line: higher values).
Panel A: Kocak-649 data set; panel B: Oberthuer-251 data set; panel C: SEQC dataset, RPM platform; Panel D: SEQC data set, custom platform.

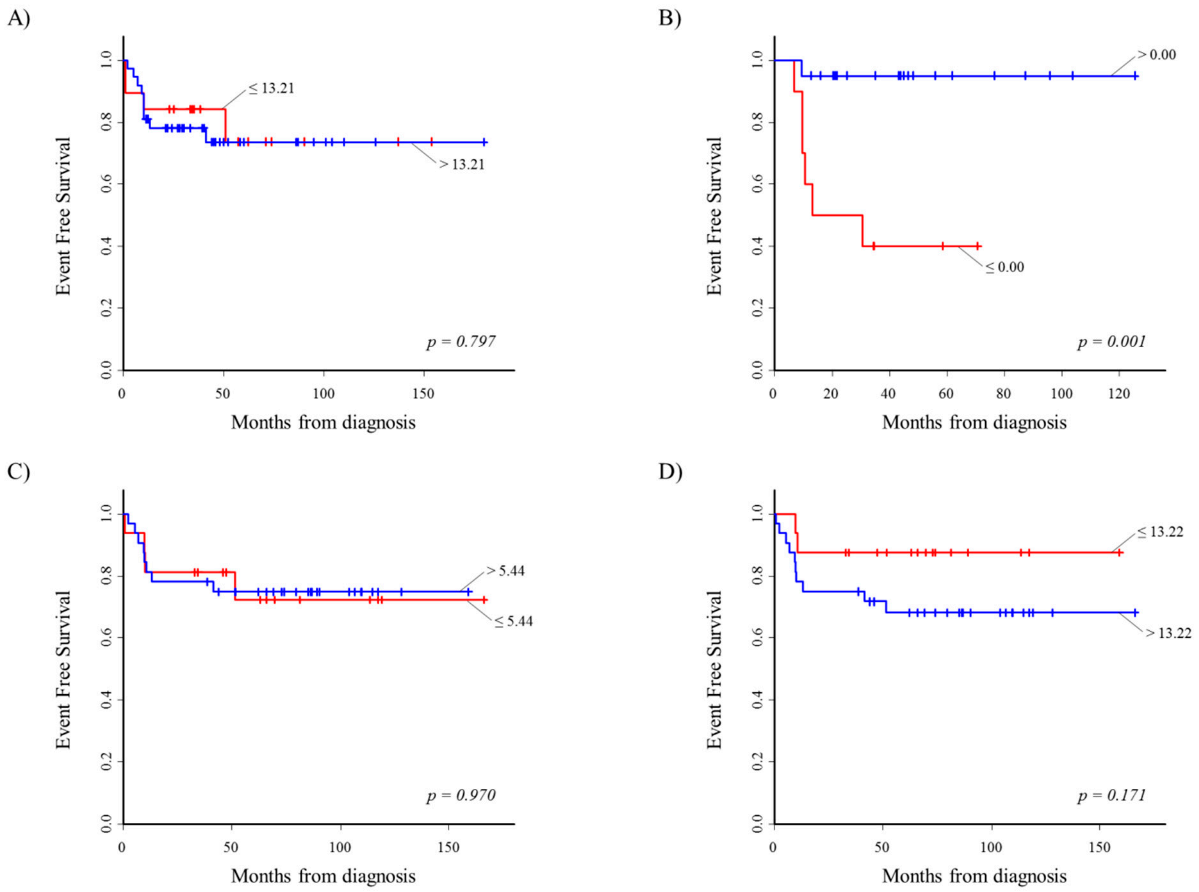

Figure S3. Event Free Survival of Stage 4S Neuroblastoma patients in relation to the expression of *RB1* gene in four microarray databases; cut-off value based on the last tertile of gene expression (red line: lower values; blue line: higher values).
Panel A: Kocak-649 data set; panel B: Oberthuer-251 data set; panel C: SEQC dataset, RPM platform; Panel D: SEQC data set, custom platform.

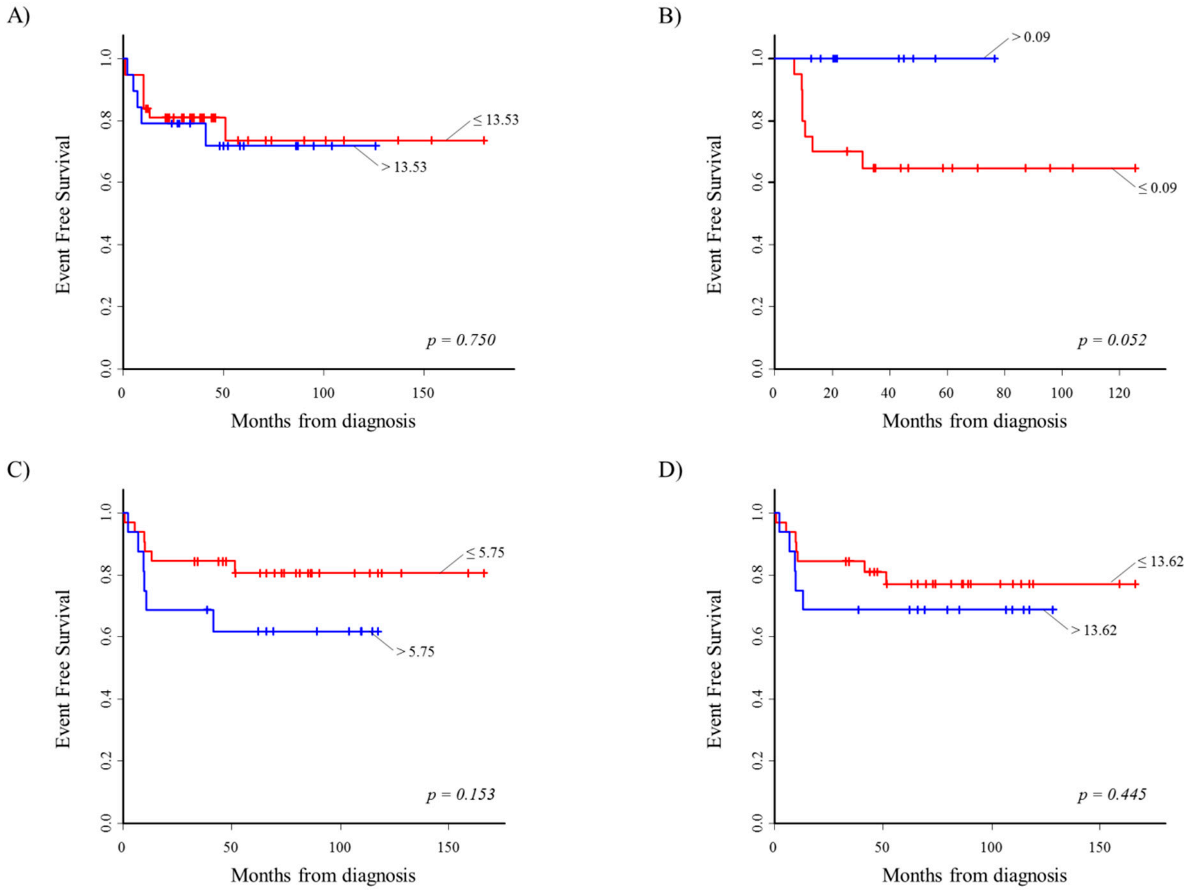

Figure S4. Forest plot of hazard ratios for the association between Event Free Survival of patients with Stage 4S Neuroblastoma and *RB1* gene expression at the following selected cut-offs: A) first tertile; B) median; C) last tertile.

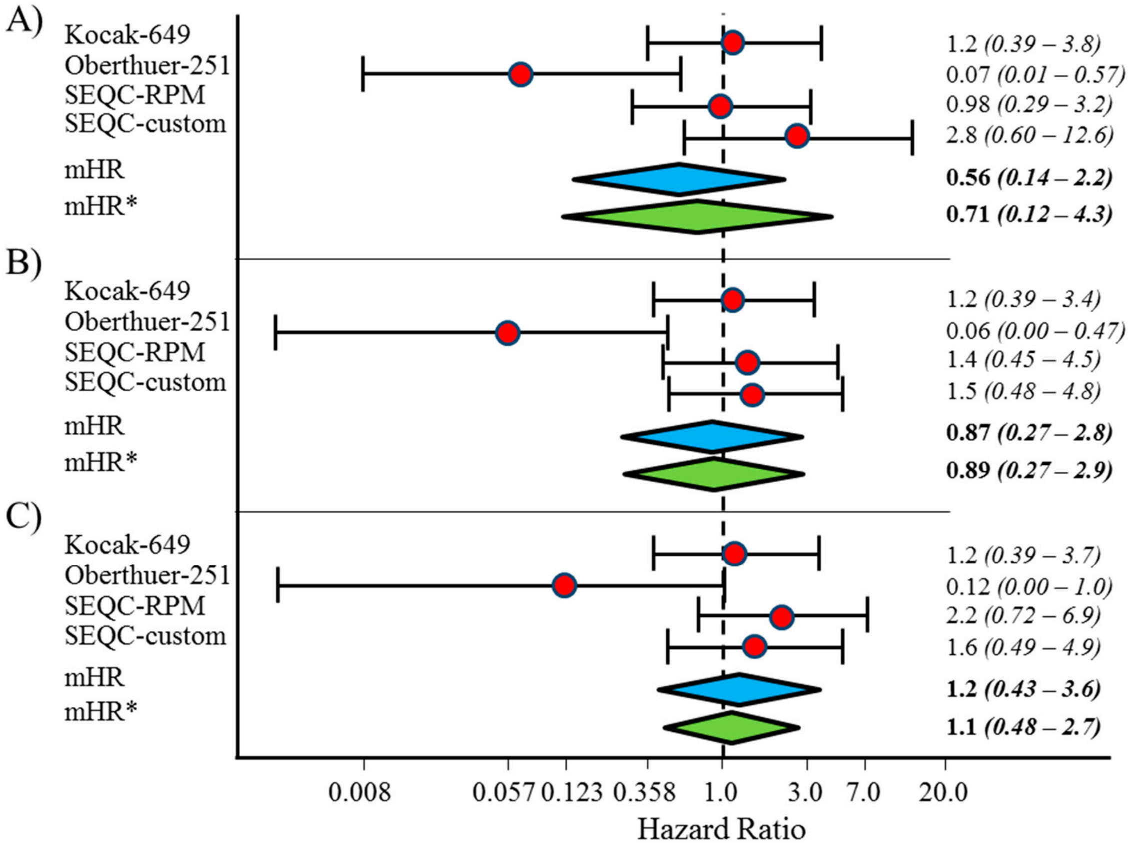

mHR: meta-analytic random effect estimates of Hazard Ratio based on Kocak, Oberthuer and SEQC RPM data sets. mHR*: meta-analytic random effect estimates of Hazard Ratio based on Kocak, Oberthuer and SEQC Custom data sets.

Figure S5. Event Free Survival of Stage 4S Neuroblastoma patients in relation to the expression of TERT gene in four microarray databases; cut-off value based on the median of gene expression (red line: lower values; blue line: higher values).
Panel A: Kocak-649 data set; panel B: Oberthuer-251 data set; panel C: SEQC dataset, RPM platform; Panel D: SEQC data set, custom platform.

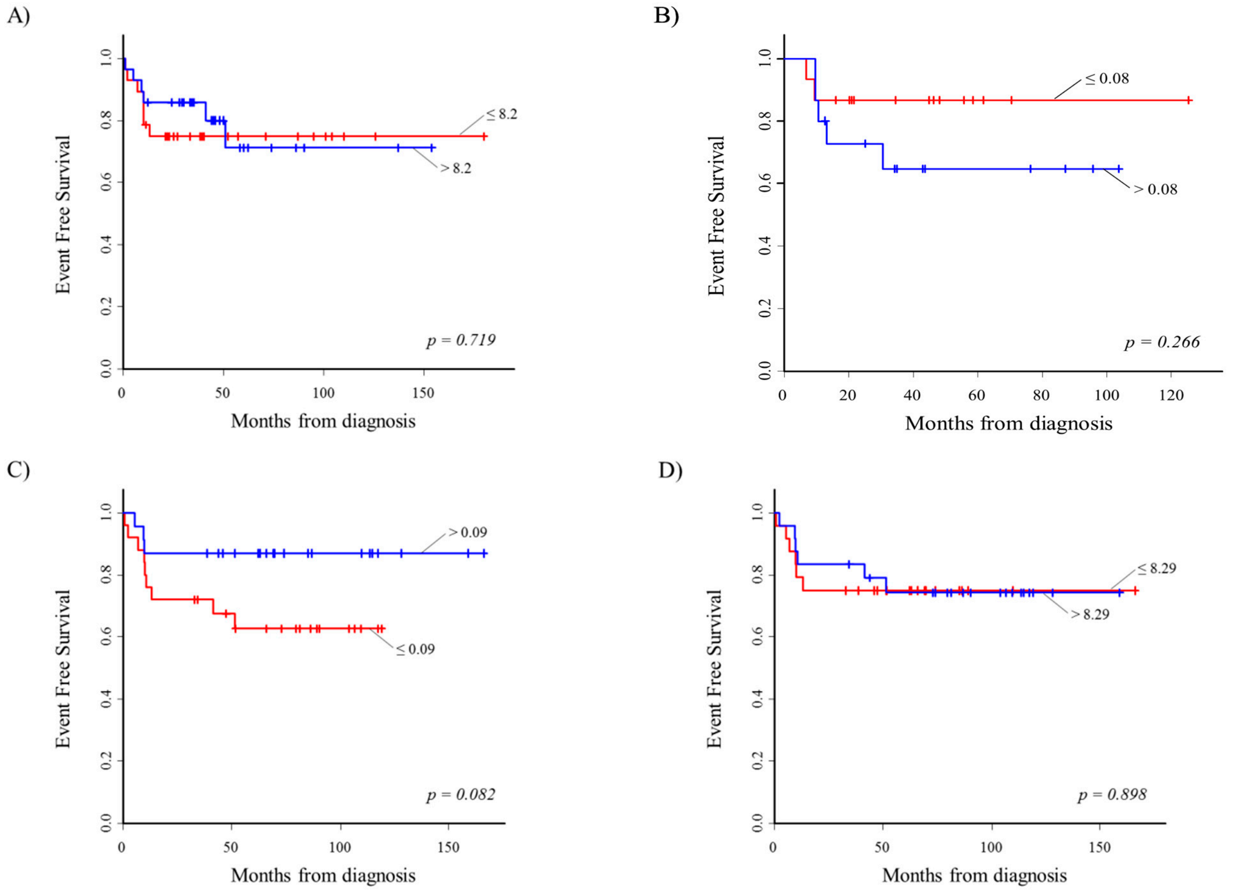

Figure S6. Event Free Survival of Stage 4S Neuroblastoma patients in relation to the expression of *TERT* gene in four microarray databases; cut-off value based on the first tertile of gene expression (red line: lower values; blue line: higher values).
Panel A: Kocak-649 data set; panel B: Oberthuer-251 data set; panel C: SEQC dataset, RPM platform; Panel D: SEQC data set, custom platform.

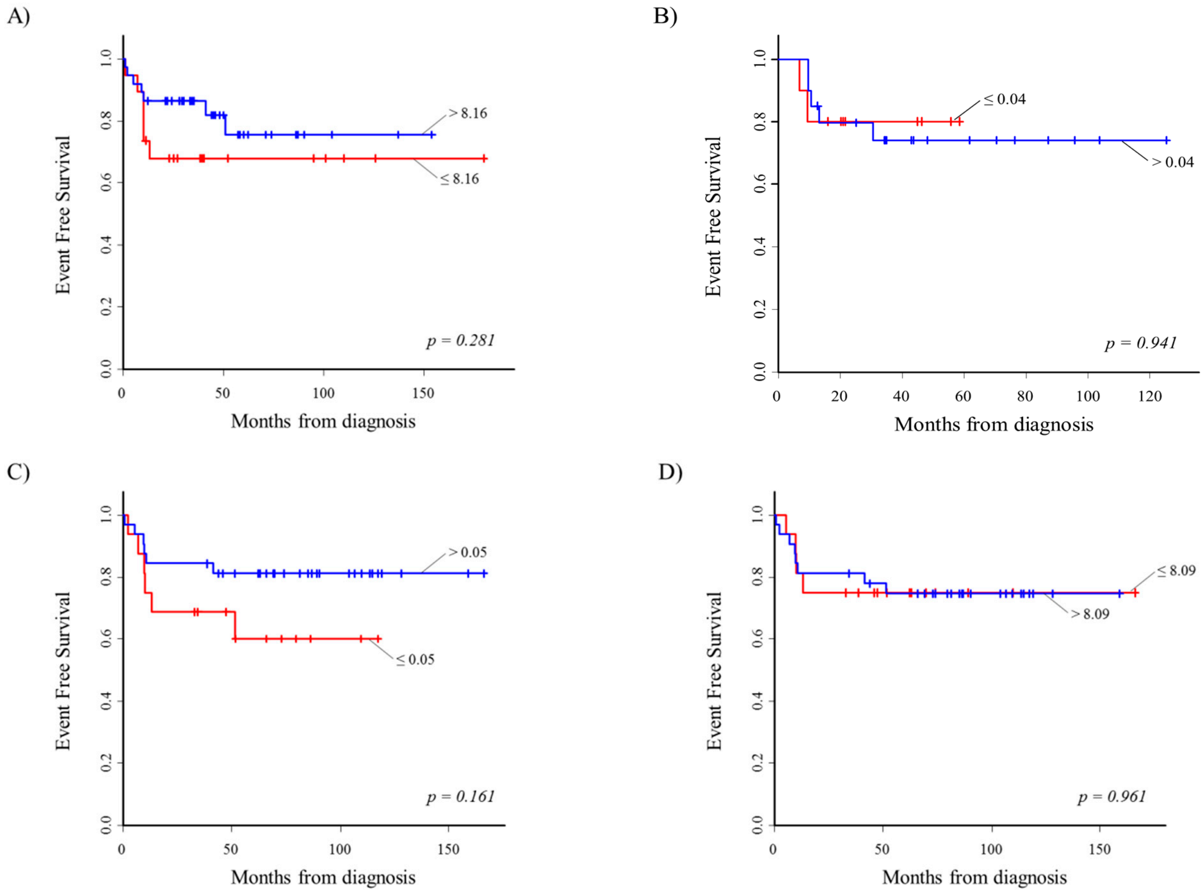

Figure S7. Event Free Survival of Stage 4S Neuroblastoma patients in relation to the expression of *TERT* gene in four microarray databases; cut-off value based on the last tertile of gene expression (red line: lower values; blue line: higher values).

Panel A: Kocak-649 data set; panel B: Oberthuer-251 data set; panel C: SEQC dataset, RPM platform; Panel D: SEQC data set, custom platform.

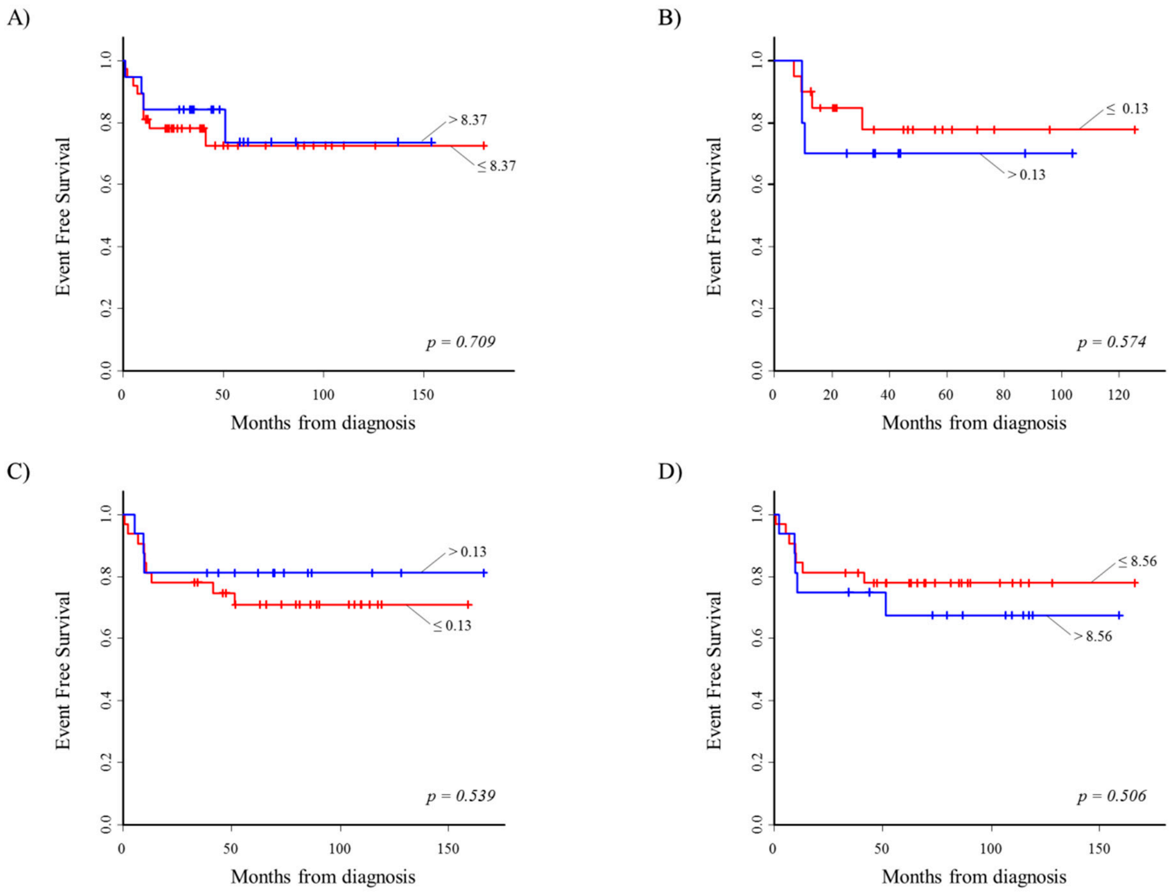

Figure S8. Forest plot of hazard ratios for the association between Event Free Survival of patients with Stage 4S Neuroblastoma and *TERT* gene expression at the following selected cut-offs: A) first tertile; B) median; C) last tertile.

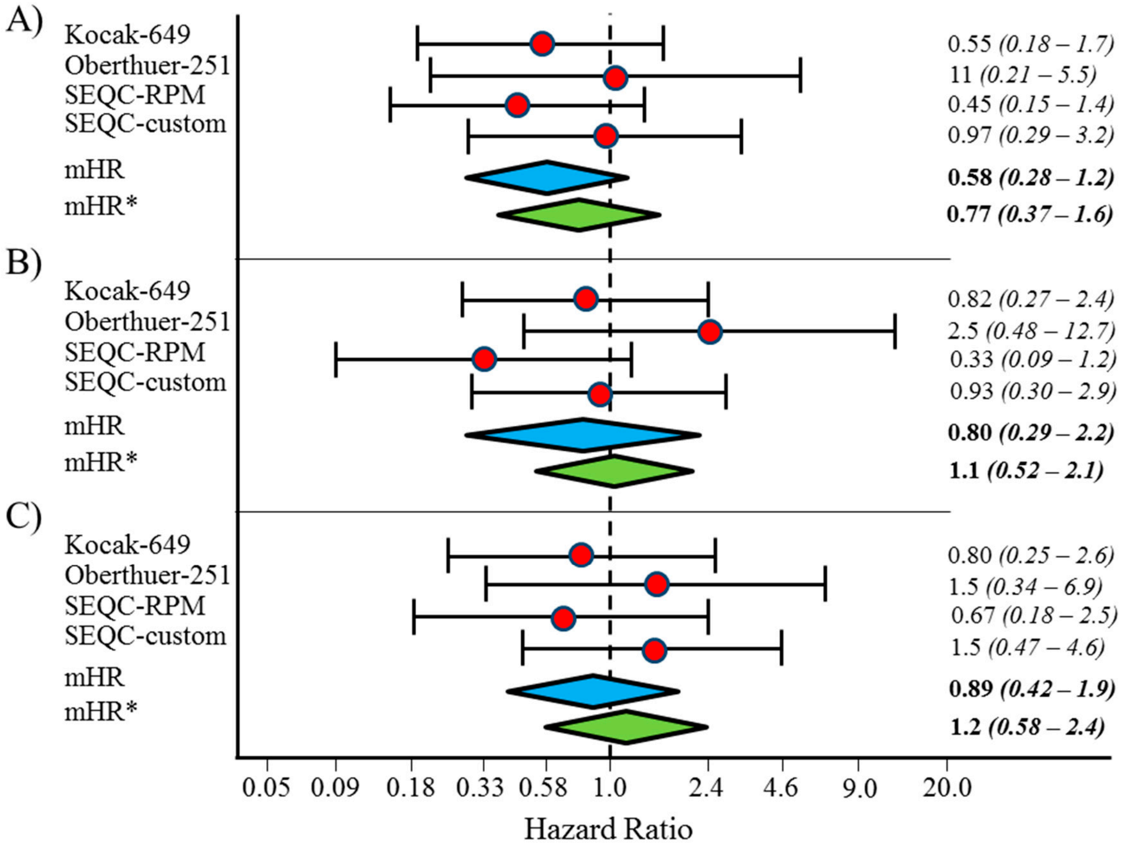

mHR: meta-analytic random effect estimates of Hazard Ratio based on Kocak, Oberthuer and SEQC RPM data sets. mHR*: meta-analytic random effect estimates of Hazard Ratio based on Kocak, Oberthuer and SEQC Custom data sets.

Figure S9. Event Free Survival of Stage 4S Neuroblastoma patients in relation to the expression of *E2F1* gene in four microarray databases; cut-off value based on the median of gene expression (red line: lower values; blue line: higher values).
Panel A: Kocak-649 data set; panel B: Oberthuer-251 data set; panel C: SEQC dataset, RPM platform; Panel D: SEQC data set, custom platform.

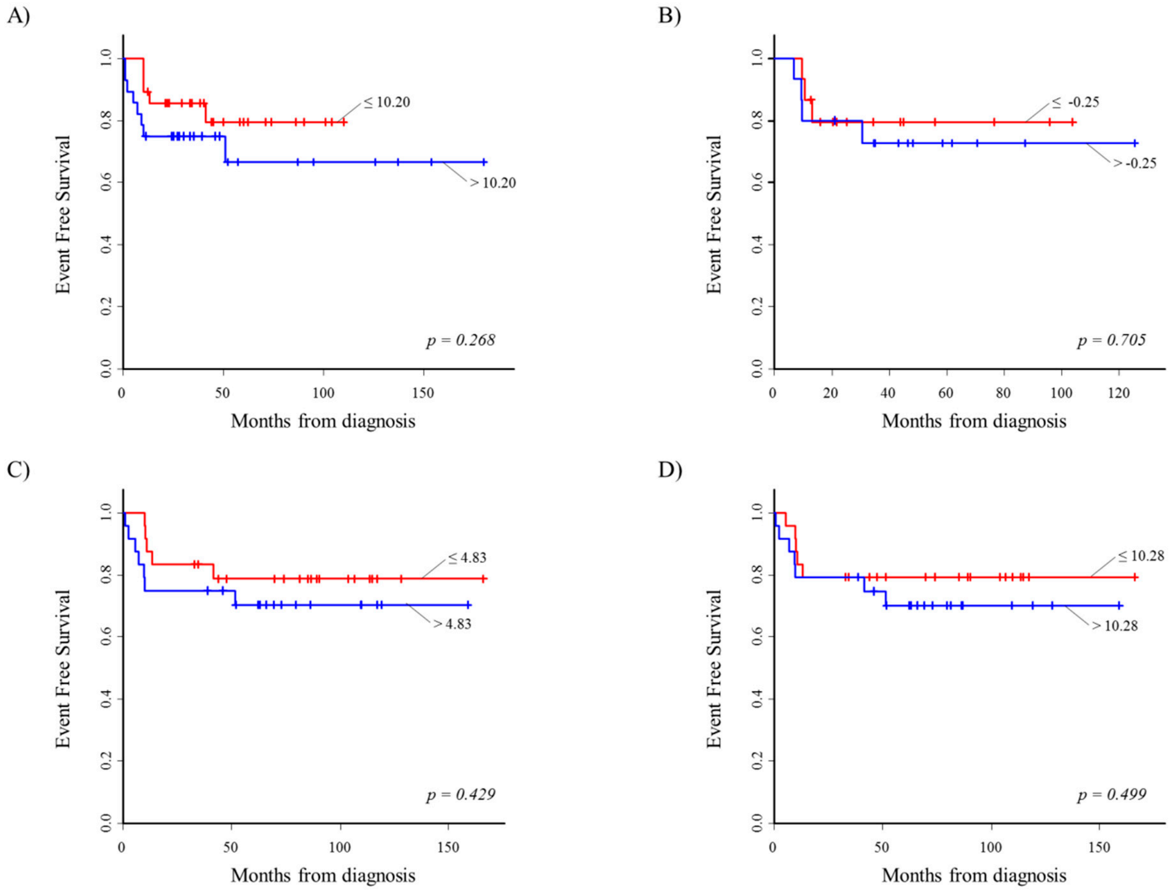

Figure S10. Event Free Survival of Stage 4s Neuroblastoma patients in relation to the expression of *E2F1* gene in four microarray databases; cut-off value based on the first tertile of gene expression (red line: lower values; blue line: higher values).
Panel A: Kocak-649 data set; panel B: Oberthuer-251 data set; panel C: SEQC dataset, RPM platform; Panel D: SEQC data set, custom platform.

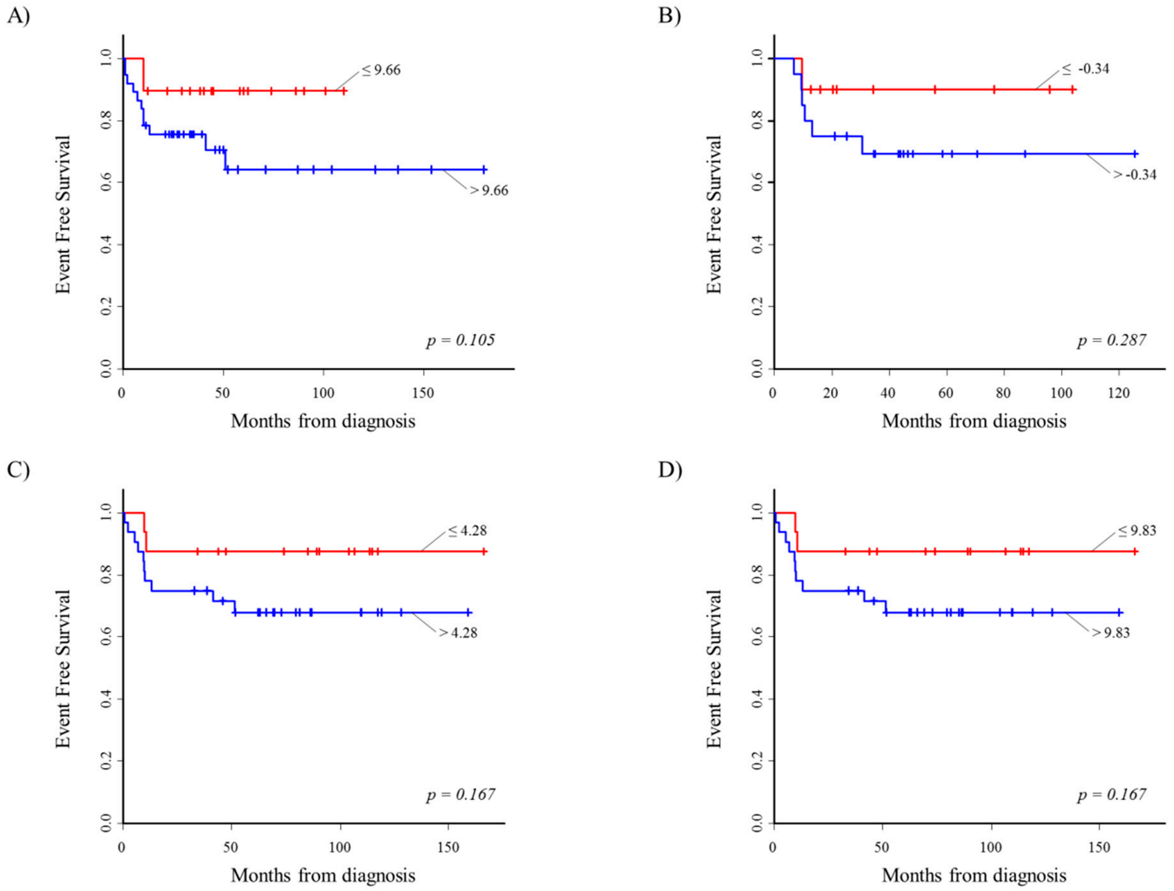

Figure S11. Event Free Survival of Stage 4S Neuroblastoma patients in relation to the expression of *E2F1* gene in four microarray databases; cut-off value based on the last tertile of gene expression (red line: lower values; blue line: higher values).
Panel A: Kocak-649 data set; panel B: Oberthuer-251 data set; panel C: SEQC dataset, RPM platform; Panel D: SEQC data set, custom platform.

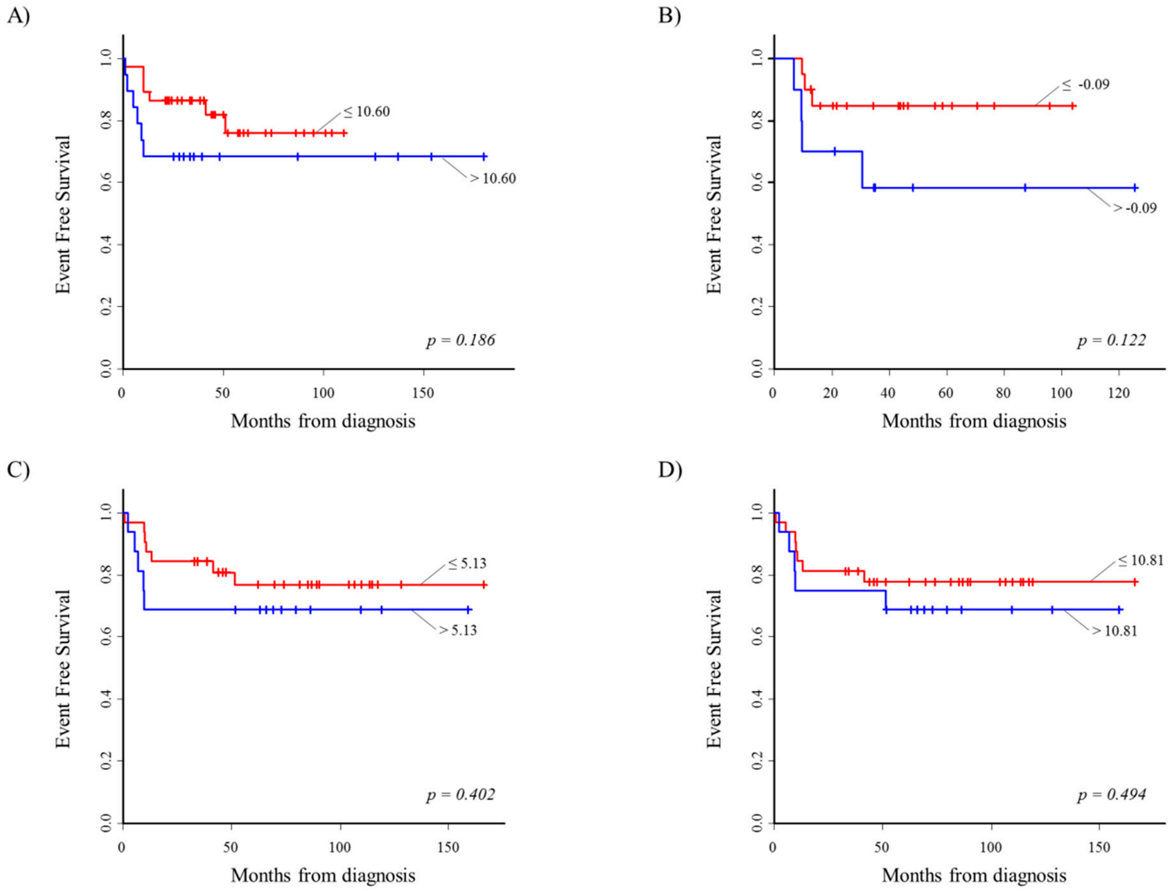

Figure S12. Forest plot of hazard ratios for the association between Event Free Survival of patients with Stage 4S Neuroblastoma and *E2F1* gene expression at the following selected cut-offs: A) first tertile; B) median; C) last tertile.

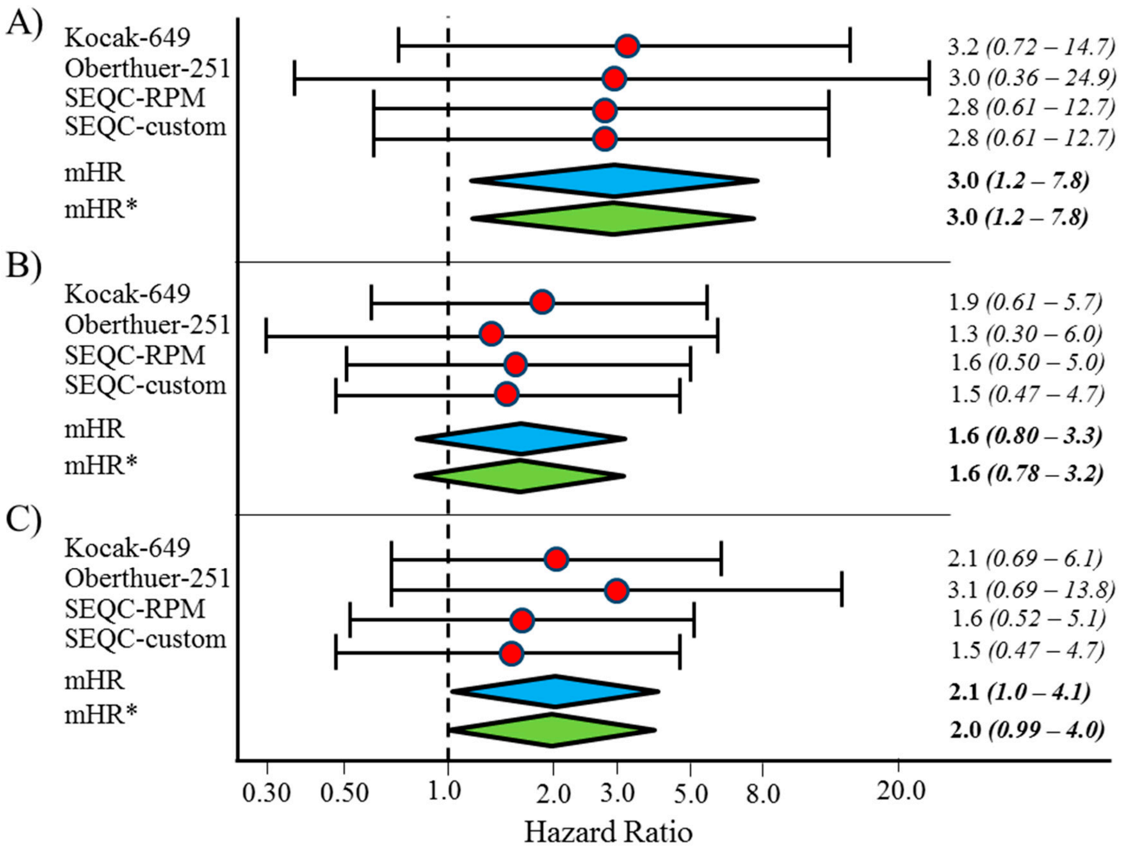

mHR: meta-analytic random effect estimates of Hazard Ratio based on Kocak, Oberthuer and SEQC RPM data sets. mHR*: meta-analytic random effect estimates of Hazard Ratio based on Kocak, Oberthuer and SEQC Custom data sets.

Figure S13. Event Free Survival of Stage 4S Neuroblastoma patients in relation to the expression of *E2F2* gene in four microarray databases; cut-off value based on the median of gene expression (red line: lower values; blue line: higher values).
Panel A: Kocak-649 data set; panel B: Oberthuer-251 data set; panel C: SEQC dataset, RPM platform; Panel D: SEQC data set, custom platform.

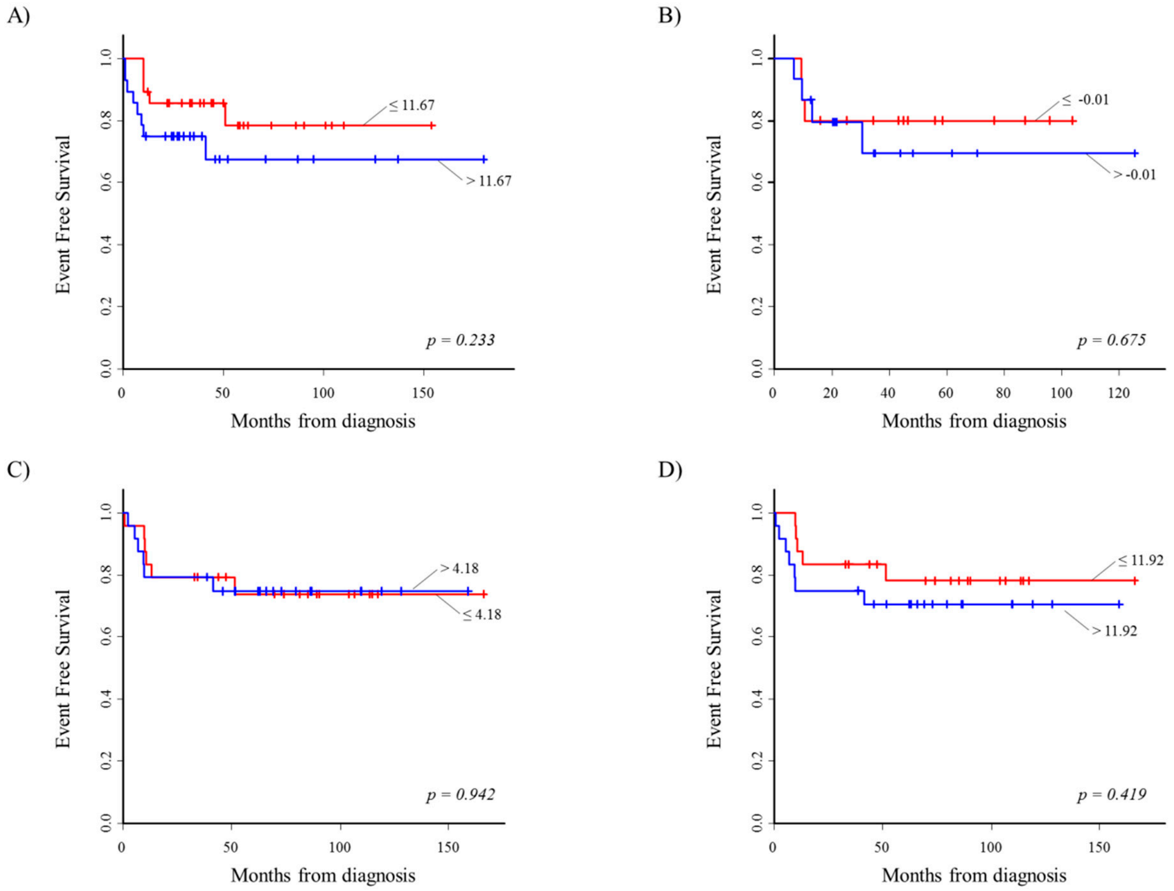

Figure S14. Event Free Survival of Stage 4S Neuroblastoma patients in relation to the expression of E2F2 gene in four microarray databases; cut-off value based on the first tertile of gene expression (red line: lower values; blue line: higher values).
Panel A: Kocak-649 data set; panel B: Oberthuer-251 data set; panel C: SEQC dataset, RPM platform; Panel D: SEQC data set, custom platform.

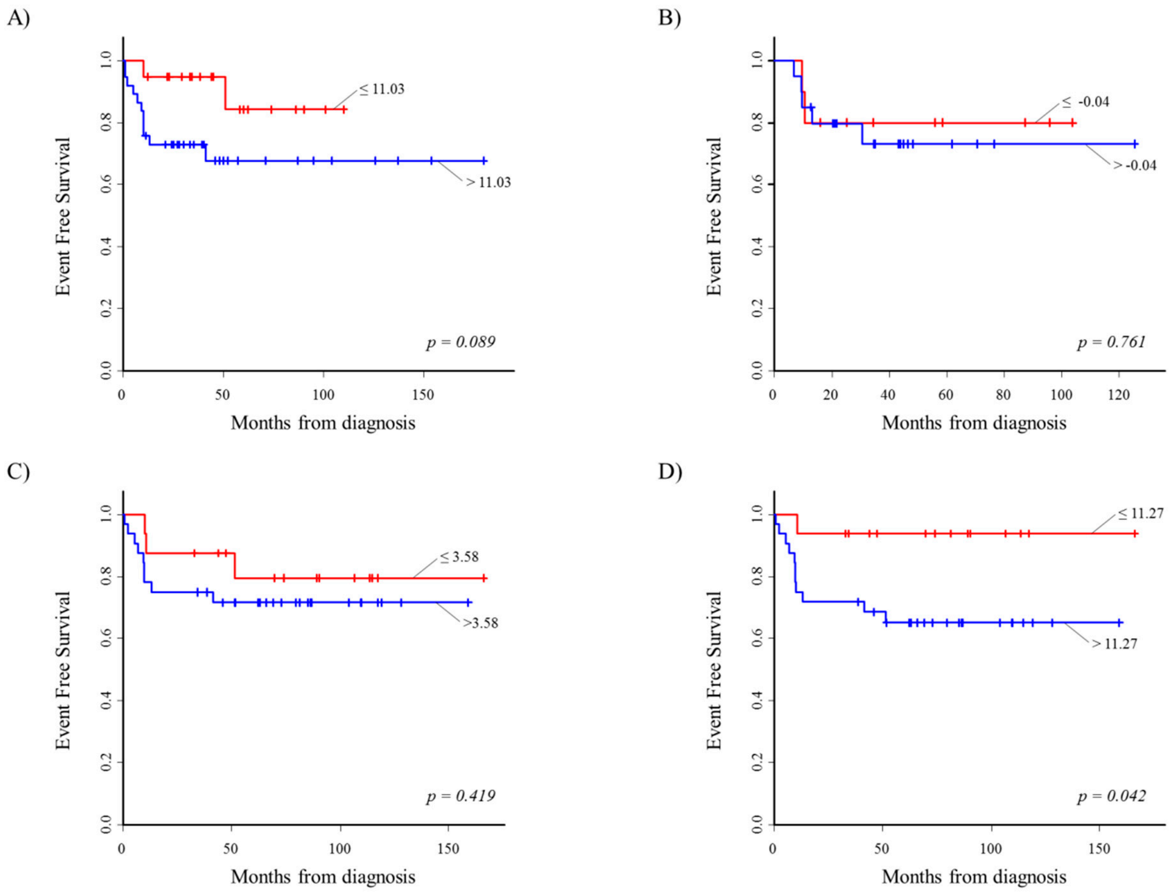

Figure S15. Event Free Survival of Stage 4S Neuroblastoma patients in relation to the expression of *E2F2* gene in four microarray databases; cut-off value based on the last tertile of gene expression (red line: lower values; blue line: higher values).
Panel A: Kocak-649 data set; panel B: Oberthuer-251 data set; panel C: SEQC dataset, RPM platform; Panel D: SEQC data set, custom platform.

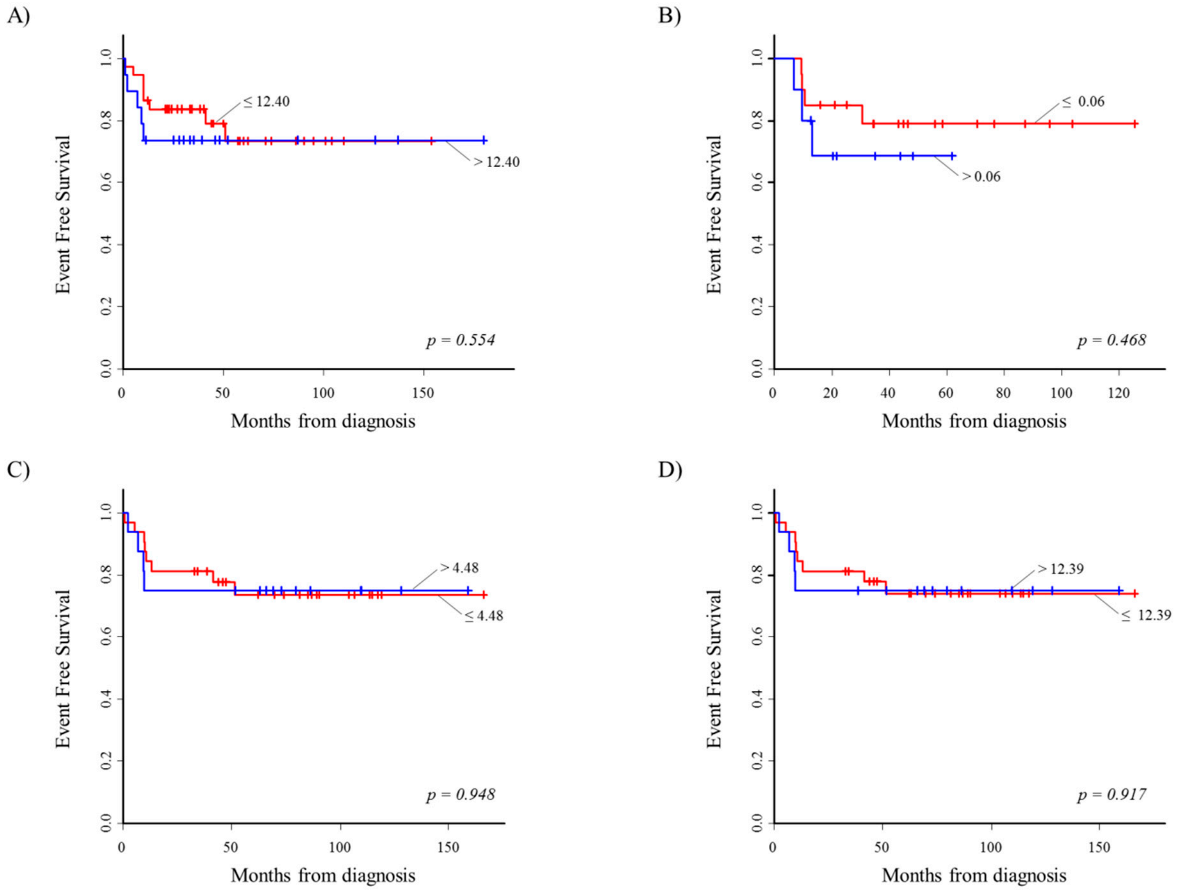

Figure S16. Forest plot of hazard ratios for the association between Event Free Survival of patients with Stage 4S Neuroblastoma and *E2F2* gene expression at the following selected cut-offs: A) first tertile; B) median; C) last tertile.

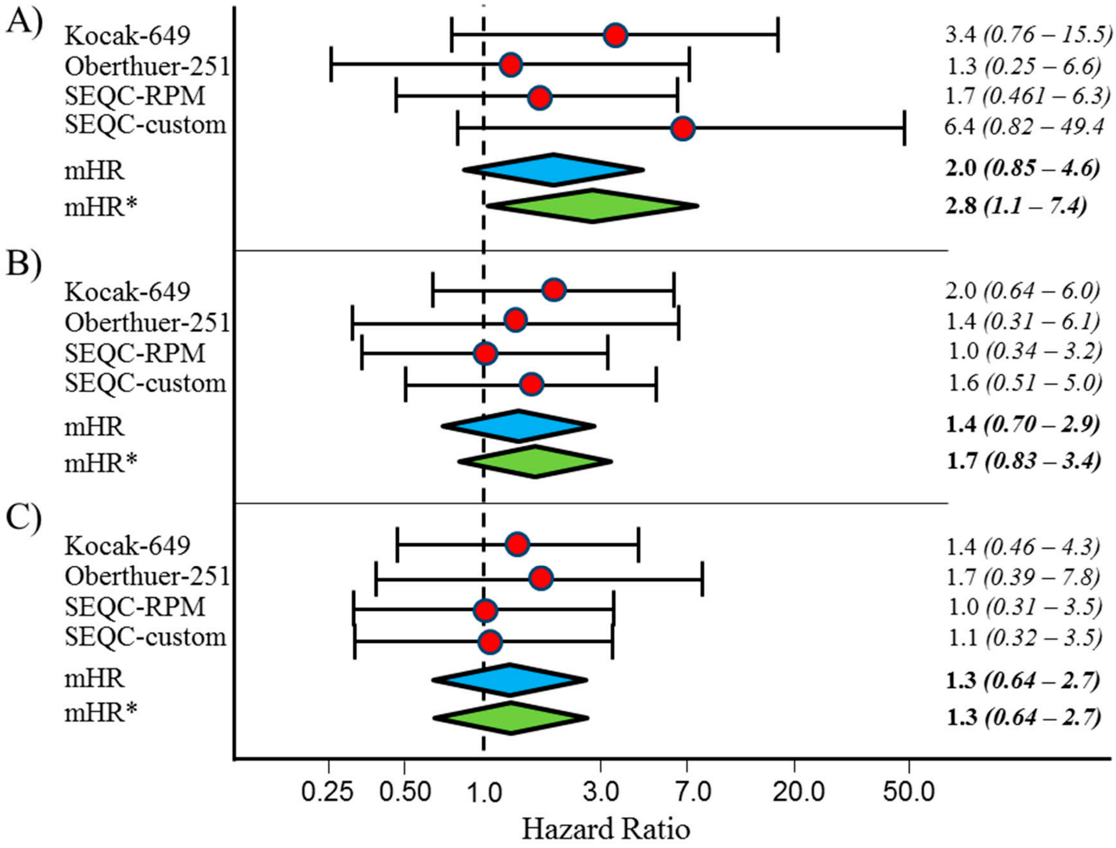

mHR: meta-analytic random effect estimates of Hazard Ratio based on Kocak, Oberthuer and SEQC RPM data sets. mHR*: meta-analytic random effect estimates of Hazard Ratio based on Kocak, Oberthuer and SEQC Custom data sets.

Figure S17. Event Free Survival of Stage 4S Neuroblastoma patients in relation to the expression of *E2F3* gene in four microarray databases; cut-off value based on the median of gene expression (red line: lower values; blue line: higher values).
Panel A: Kocak-649 data set; panel B: Oberthuer-251 data set; panel C: SEQC dataset, RPM platform; Panel D: SEQC data set, custom platform.

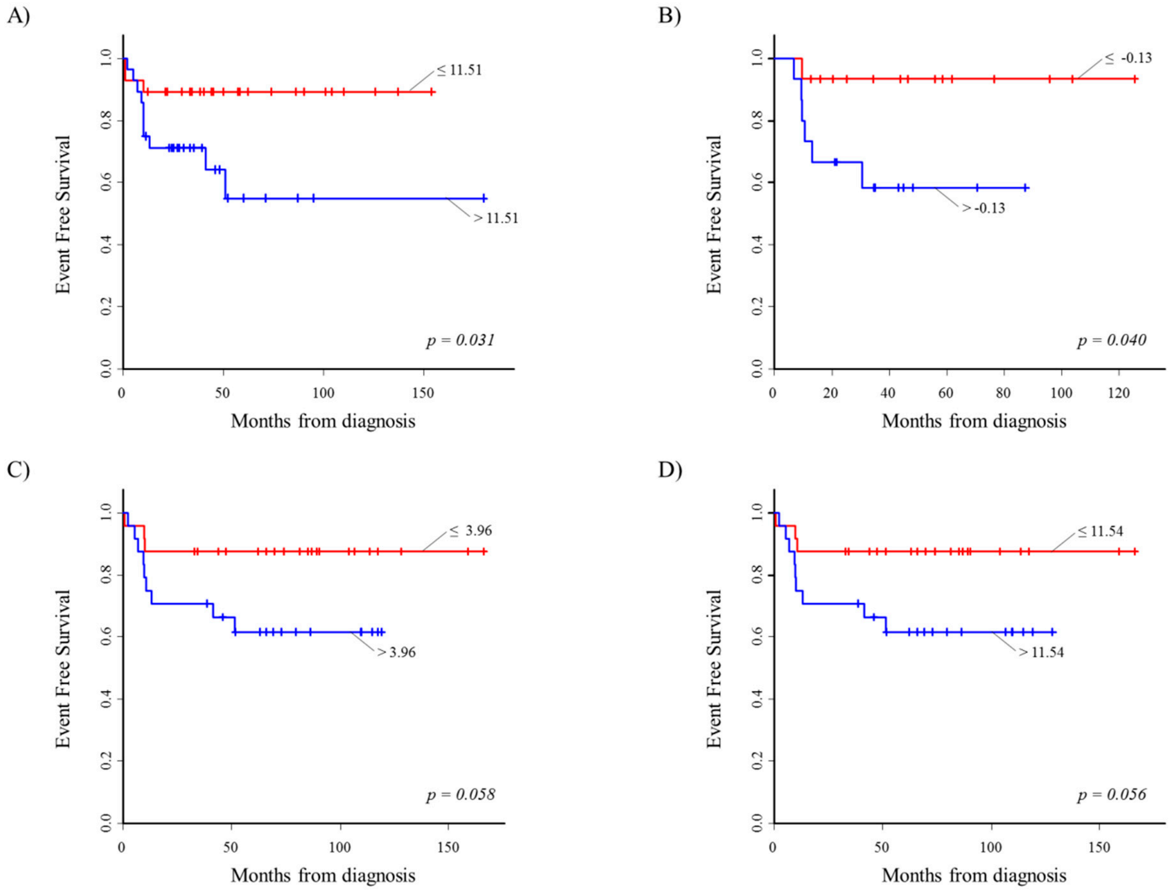

Figure S18. Event Free Survival of Stage 4S Neuroblastoma patients in relation to the expression of *E2F3* gene in four microarray databases; cut-off value based on the first tertile of gene expression (red line: lower values; blue line: higher values).
Panel A: Kocak-649 data set; panel B: Oberthuer-251 data set; panel C: SEQC dataset, RPM platform; Panel D: SEQC data set, custom platform.

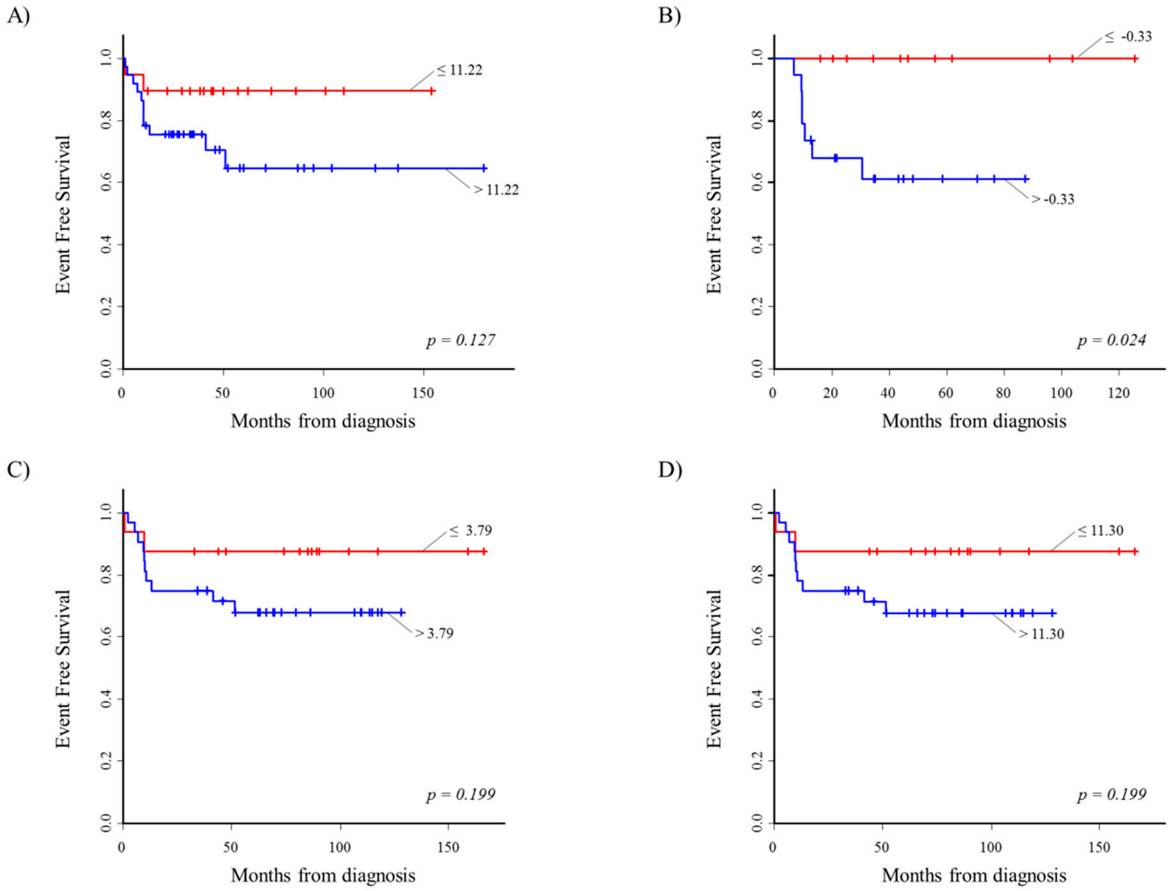

Figure S19. Event Free Survival of Stage 4S Neuroblastoma patients in relation to the expression of *E2F3* gene in four microarray databases; cut-off value based on the last tertile of gene expression (red line: lower values; blue line: higher values).
Panel A: Koca-649 data set; panel B: Oberthuer-251 data set; panel C: SEQC dataset, RPM platform; Panel D: SEQC data set, custom platform.

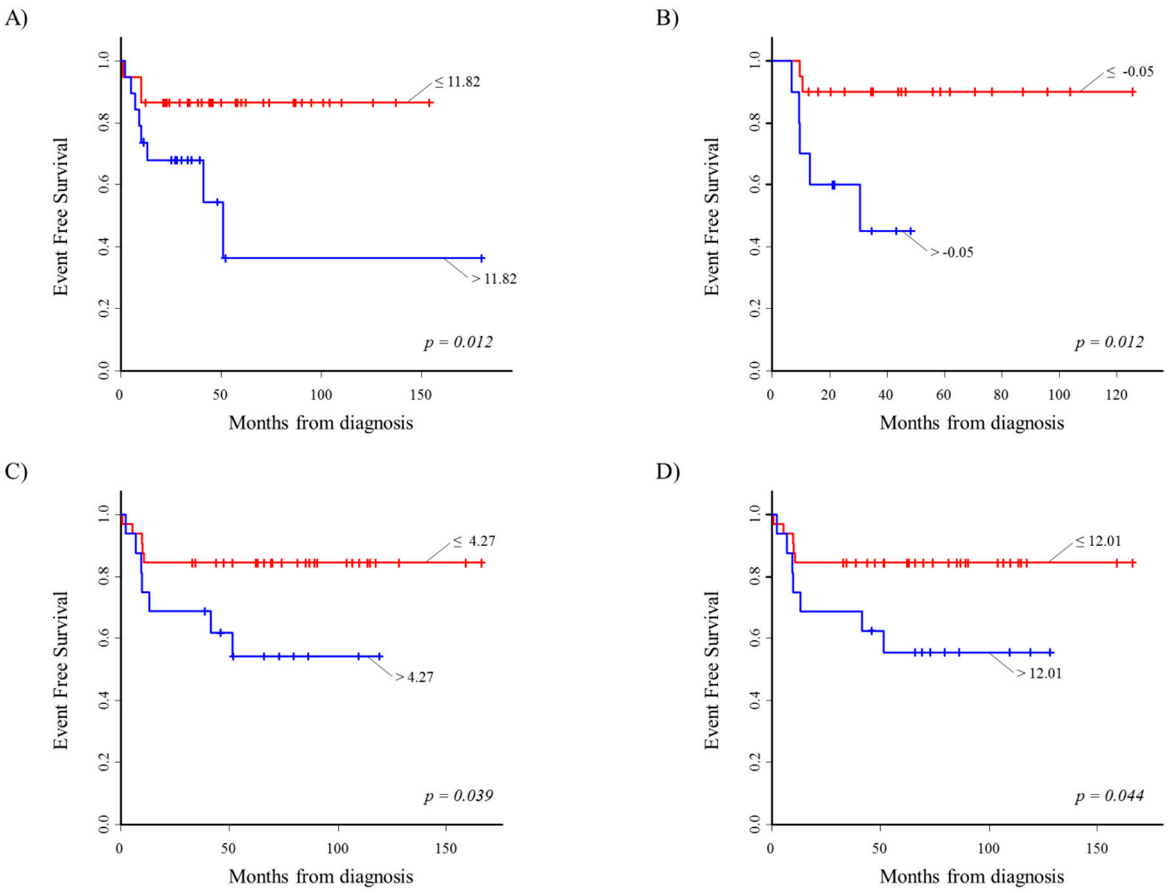

Figure S20. Forest plot of hazard ratios for the association between Event Free Survival of patients with Stage 4S Neuroblastoma and *E2F3* gene expression at the following selected cut-offs: A) first tertile; B) median; C) last tertile.

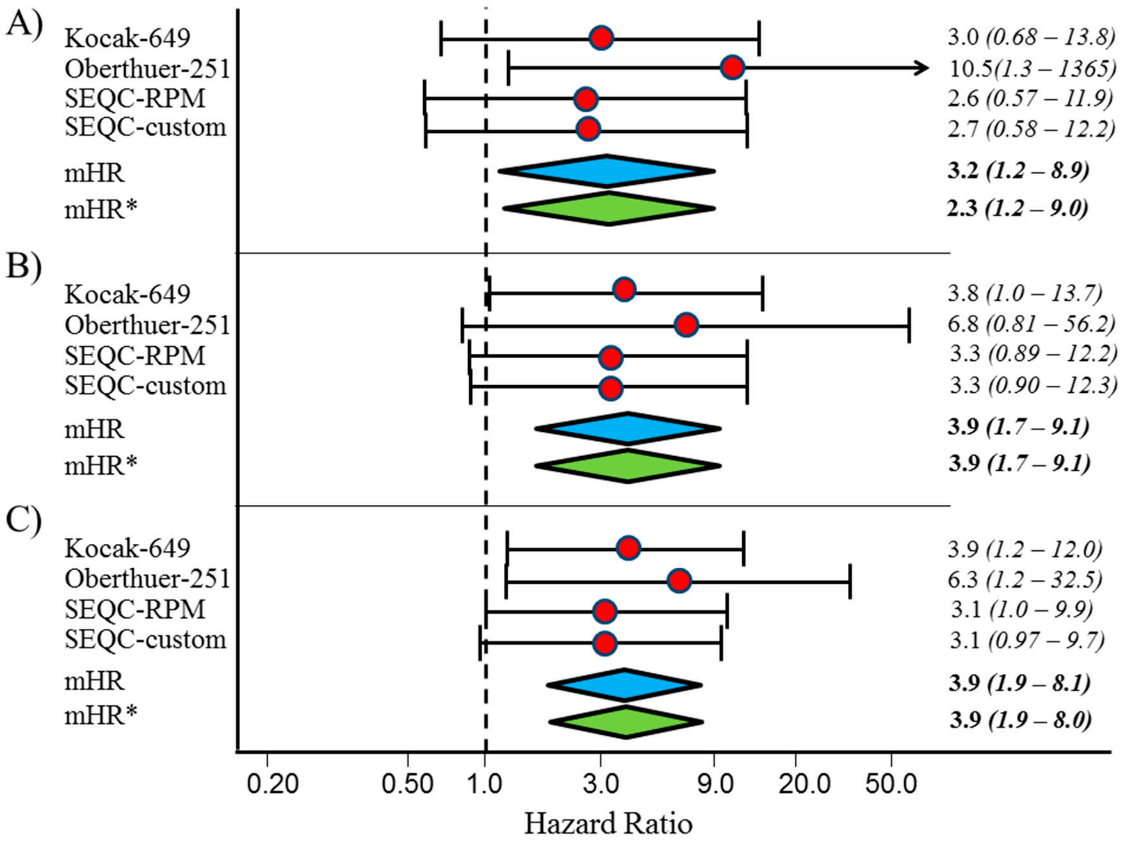

mHR: meta-analytic random effect estimates of Hazard Ratio based on Kocak, Oberthuer and SEQC RPM data sets. mHR*: meta-analytic random effect estimates of Hazard Ratio based on Kocak, Oberthuer and SEQC Custom data sets.

Table S1. Analysis of E2F3 protein in 38 primary neuroblastoma tissue sections.

Patient	Stage	Protocol	*MYCN* amplified	Relapse	Relapse site	E2F3⁺cells (%)
NB1	4S	Infant	N	N	-	3
NB2	4S	Infant	N	N	-	5
NB3	4S	Infant	N	N	-	6
NB4	4S	Infant	N	N	-	18
NB5	4S	Infant	N	N	-	4
NB6	4S	Infant	N	Y*	bone marrow, liver	90
NB7	4S	Infant	N	**Y***	lung	80
NB8	4S	Infant	N	N	-	16
NB9	4S	Infant	N	**N**	-	2
NB10	4S	Infant	N	**N**	-	10
NB11	4S	Infant	N	N	-	8
NB12	4S	Infant	N	**Y**	metastatic	87
NB13	4S	Infant	N	Y	distant and local	90
NB14	4S	Infant	N	N		5
NB15	4S	Infant	N	N		4
NB16	4S	Infant	N	N		11
NB17	4S	Infant	N	N		3
NB18	4S	Infant	N	N		6
NB19	4S	Infant	N	N		14
NB20	4S	Infant	N	N		7
NB21	4S	Infant	N	N		5
NB22	4S	Infant	N	N		4
NB23	4S	Infant	N	N		**9**
NB24	4S	Infant	N	Y	metastatic	90
NB25	4S	Infant	N	Y	local	80
NB26	4S	Infant	N	Y	metastatic	90
NB27	4S	Infant	N	N		12
NB28	4S	Infant	N	N		4
NB29	4S	Infant	N	N		7
NB30	4S	Infant	N	N		3
NB31	4S	Infant	N	N		8
NB32	4S	Infant	N	Y	metastatic	78
NB33	4S	Infant	N	Y	distant and local	90
NB34	4S	Infant	N	Y*	central nervous system	80
NB35	4S	Infant	N	Y	metastatic	80
NB36	4S	Infant	N	Y	metastatic	90
NB37	4S	Infant	N	Y	distant and local	90
NB38	4S	Infant	N	Y*	bone marrow, bones	90

Y= yes; N= not; * tumor progression to high risk stage 4
